# The State of Social Media Policies in Higher Education

**DOI:** 10.1371/journal.pone.0127485

**Published:** 2015-05-27

**Authors:** Jeffrey Pomerantz, Carolyn Hank, Cassidy R. Sugimoto

**Affiliations:** 1 School of Information and Library Science, University of North Carolina at Chapel Hill, Chapel Hill, North Carolina, United States of America; 2 School of Information Sciences, University of Tennessee, Knoxville, Knoxville, Tennessee, United States of America; 3 School of Informatics and Computing, Indiana University Bloomington, Bloomington, Indiana, United States of America; Illinois Institute of Technology, UNITED STATES

## Abstract

This paper presents an analysis of the current state of development of social media policies at institution of higher education. Content analysis of social media policies for all institutions listed in the Carnegie Classification Data File revealed that less than one-quarter of institutions had an accessible social media policy. Analysis was done by institution and campus unit, finding that social media policies were most likely to appear at doctorate-granting institutions and health, athletics, and library units. Policies required that those affiliated with the institution post appropriate content, represent the unit appropriately, and moderate conversations with coworkers and external agencies. This analysis may inform the development and revision of social media policies across the field of higher education, taking into consideration the rapidly changing landscape of social media, issues of academic freedom, and notions of interoperability with policies at the unit and campus levels.

## Introduction

Geoffrey Miller tweeted something that he regrets.

Geoffrey Miller is hardly unique in this. He is certainly not the only person to ever regret a tweet or a post to Facebook or some other communication on some other social media platform [1]. The difference is that Geoffrey Miller nearly lost his job because of his tweet.

On 2 June 2013, Geoffrey Miller tweeted the following: “Dear obese PhD applicants: if you didn't have the willpower to stop eating carbs, you won't have the willpower to do a dissertation. #truth”. As might have been expected, this led to an immediate and hostile backlash on Twitter. At the time, Miller was a visiting professor at New York University, on leave from the University of New Mexico. This tweet was ill-conceived any way you look at it, but by directly addressing PhD applicants, Miller placed himself in an institutional context and evoked his role as a faculty member. Miller quickly apologized on Twitter and made his account private [2] [3], and later told his department chair at UNM that the tweet “was part of a research project” [4]. At the time of this writing, both universities had conducted disciplinary inquiries into the matter, and Miller had been formally censured by UNM [5] [6].

Many public figures have run into problems over what they have posted to a social media platform. During the 2012 Summer Olympics, for example, two athletes were expelled from the Games: Voula Papachristou, a member of the Greek Olympic team, and Michel Morganella, a member of the Swiss team, for tweeting racist comments [7] [8]. These incidents were public and high-visibility specifically because they happened on Twitter. It was possible for the International Olympic Committee (IOC) to expel Papachristou and Morganella because a set of guidelines exists that dictates appropriate conduct on social media during the Olympics.

No such guidelines existed at either New York University or the University of New Mexico, which must have made it difficult for the administrations of those universities to determine an appropriate course of action with regard to Geoffrey Miller. Indeed, instead of addressing Miller's original tweet, both universities focused on Miller's later claim that the tweet was research. It's easy to see why: NYU and UNM, like most universities in the United States, have institutional review boards and thorough sets of policies governing the conduct of research. If the claim of tweeting in the name of research turned out to be false – as UNM ruled it to be [3] – then there would be clear policies to fall back on. In the absence of policies governing the use of social media, however, these universities' administrations lacked justification for investigating Miller's behavior.

The catalog of incidences of ill-advised academic tweeting continues to grow: recent stories that have attracted the attention of the popular and academic presses have included Gloria Gadsen’s suspension from East Stoudsburg University after posting threatening Facebook posts, and the outrage from the National Rifle Association after University of Kansas professor David Gruth’s inflammatory post about the Navy Yard shooting. The creation of university policies governing the use of social media are a reasonable reaction to these highly publicized tweets. However, as is clear in the case of the University of Kansas situation, such policies are difficult to construct in a way that suits all stakeholders and protects academic freedom [9].

This paper presents a survey of social media policies at institution of higher education. As with any new policy arena, this landscape is shifting rapidly. This paper provides a description of the current degree to which institutions and campus units have developed social media policies, and a detailed look at the contents of existing policies. It is the authors’ intention that this analysis can inform the development and revision of social media policies, as well as set a benchmark against which future developments can be measured.

## Literature Review

An extensive body of literature exists on various aspects of social media. The Pew Internet & American Life Project is a leader in this area—conducting and making available a wide range of studies on the topic of social networking [10]. There is also an active research community addressing the role of social media in such diverse areas as the lives of teenagers [11], personal privacy [12], and political movements [13].

Literature on social media policies, however, has predominantly appeared in the business trade press. This work, as might be expected, tends to be more pragmatic: why organizations should have social media policies [14], how to write these policies [15], and how to leverage social media for the benefit of the organization [16] [17]. Indeed, developing social media policies for corporations, and the appropriate scope and content of those policies, is such a significant issue that no less than the United States Federal National Labor Relations Board has issued a report analyzing legal cases in which employers' social media policies came under question, and providing guidance for developing a legally compliant social media policy [18]. Legal necessity may also be the driver in the analysis of the role of social media in the health education environment (e.g., [19] [20] [21]). The extant literature in this area focuses on how social networking has been used and the potential ethical dilemmas of this use (e.g., [22]).

To date, however, there has only been one study that analyzes the content of social media policies themselves. That study [23] [24] was an analysis of 46 social media policy documents that were publicly available online. Boudreaux analyzes corporate social media policies according to several criteria, including tone, separation of personal and official uses, guidelines regarding the use of specific social media applications, and links to other relevant organizational policies. Boudreaux [24] found that “social media policies tend to evolve through three distinct stages”: Mitigation, Information, and Differentiation. Policies focused on mitigation are concerned with risk and protecting the organization, and all tend to look similar, containing recommendations such as to "be authentic” and to respect copyright. Informational policies start to diverge, as organizations learn to use social media to communicate their unique values, goals, and culture; these policies contain information about, for example, the types of personal data that the organization collects and maintains, and links to other relevant organizational policies. Policies in the differentiation phase provide “thoughtful guidance that empowers employees to differentiate the organization in the market” (p. 283). Most of the social media policies that Boudreaux analyzed were from corporations, though some were from county- and state-level governments, and branches of the US military. Unfortunately, none were from institutions of higher education. Indeed, as of this writing, there seem to be no studies of social media policies from institutions of higher education.

## Methods

In the early 1970s, the Carnegie Foundation for the Advancement of Teaching developed what has come to be the most commonly used classification system for institutions of higher education worldwide: the Carnegie Classification of Institutions of Higher Education. Since then, the Carnegie Classification has undergone several revisions; the most recent and most sweeping of which was released in 2005 [25]. The Carnegie Foundation provides a data file for download, containing the names and locations of all accredited, degree-granting colleges and universities in the United States, as well as their classifications (classifications.carnegiefoundation.org/resources/). This data file was used as the starting point for this study, as the most complete list available of institutions of higher education in the United States.

The Carnegie Classifications Data File lists a total of 4,635 institutions, of 33 types. The Carnegie Commission on Higher Education has developed the “Basic Classification,” which divides institutions into 6 categories, each of which has several subcategories, except for Tribal Colleges, which has no subcategories. For the purposes of our analyses – and with apologies to our readers at Tribal Colleges – we collapsed Special Focus Institutions and Tribal Colleges into one category, since there are only 31 Tribal Colleges out of 4,635 institutions listed in the Carnegie Classifications Data File, and only 4 that we found to have social media policies. The number of institutions in each top-level category in the Data File is as follows:

- Doctorate-granting Universities: 284
- Master's Colleges and Universities: 651
- Baccalaureate Colleges: 749
- Associate's Colleges: 1,692
- Special Focus Institutions / Tribal Colleges: 775
- Unclassified / Data not available: 484

Amazon's Mechanical Turk was used to collect and analyze data for this study, in three stages, between August 2012 – July 2013. In conducting this research and publishing this manuscript, the authors were careful to adhere to the terms of the Amazon Mechanical Turk Participation Agreement [26]. While some have raised concerns about ethical issues involved in crowdsourcing work [27], the quality of work produced in this way has been established in a variety of fields [28] [29] [30], and research has even been conducted to identify techniques for how to obtain quality work [31]. The authors took into consideration the ethical concerns raised by others about Mechanical Turk, and attempted to ensure that Turk workers were reasonably well paid for completing our tasks. Mechanical Turk computes the Effective Hourly Rate of workers based on the payment per assignment that the task Requester specifies, and the average time per assignment taken by the worker. It is difficult to predict in advance exactly how long a task will take a worker, but we managed to achieve an Effective Hourly Rate of between $8–16 for all three of our stages.

### Stage 1: Collecting URLs

The first stage of use of Mechanical Turk was to collect the URLs for the websites of the institutions listed in the Carnegie Classifications Data File. This data collection was conducted between 17–23 August 2012. This was a straightforward task: given the name, city, and state of an institution (from the Carnegie Classifications Data File), the Turk worker was asked to provide the URL of the institution's official website.

Websites were found for 99% of the institutions in the Carnegie Classifications Data File had websites (n = 4,581). The researchers had expected that figure to be 100%, given the ubiquity of the web in mid-2013, when this study was conducted. Judging by their names (because there was not much else to go on, not having a website to consult), most of the institutions that did not have websites were yeshivas (Jewish religious institutions that produce rabbis, more or less the equivalent of seminaries).

Many of the institutions listed in the Carnegie Classifications Data File are members of larger systems – including all of the authors' own institutions. Each campus in a university system is unique, however, with its own research and teaching strengths. This differentiation makes it clear that, for example, UNC-Chapel Hill and UNC-Asheville are different institutions, and not merely instances of a “franchise.” This is not the case for all institutions in the Carnegie Classifications Data File: the data file lists institutions such as the ITT Technical Institute and the University of Phoenix, where there is little differentiation between campus locations – though, to be fair, these institutions do not intend for there to be such differentiation. The Carnegie Classifications Data File lists all of the many locations of such “franchise” institutions, even though they are not truly unique except in their geographic locations.

Fortunately, there was an easy way to identify most of these franchise institutions in the Carnegie Classifications Data File: their URLs share a common domain. For example, all campuses of the ITT Technical Institute share the domain itt-tech.edu. (More differentiated institutions of course have unique URLs: unc.edu is UNC-Chapel Hill, for example, while unca.edu is UNC-Asheville.) This method did not identify all franchise institutions, but it identified most; the rest were identified manually by the researchers.

Prior to the second stage of data collection, all URLs for franchise institutions were collapsed into one single URL for the umbrella institution. This was done because the policies at these institutions are uniform across all locations – unlike in many state university systems. After this collapsing, 3,620 URLs remained.

### Stage 2: Collecting Social Media Policies

The second stage of use of Mechanical Turk was to collect the URLs of institutions' social media policies or guidelines, if any. This data collection was conducted between 28 September – 18 December 2012. The URLs identified in the previous stage were used to construct Google searches on institutions' websites for policies that address social media. Of course, not all institutions of higher education have separate policies that address social media; some institutions integrate social media guidelines into other policy documents, such as faculty and student handbooks. Google searches were therefore constructed so as to retrieve documents of all of these types. While Google has its limitations, the variability in search tools implemented on different institutions' websites is too great to provide consistent results. Thus Google was used to provide consistency across searches. The “search within a site” feature of Google was employed, making use of these institutions' URLs, to supplement keyword searching. The Google search results were embedded in the task page seen by Mechanical Turk workers. Searches followed this pattern:

(“social media” OR “social networking”) (policy OR guidelines OR handbook) site:unc.edu

Each search was provided to three Turkers. Google personalizes search results for individual users [32], but this was not an issue here, as the “user” doing the searching was Mechanical Turk; therefore the results from each search were identical for all three Turkers. Searches were provided to multiple Turkers in order to ensure the identification of the maximum number of documents that could be considered social media policies or guidelines. This approach ran the clear risk of false positives, where some Turkers would identify documents that were not social media policies or guidelines. However, the researchers considered that less of a risk than false negatives: we decided that it would be easier to screen out documents that were not social media policies or guidelines, than it would be to identify ones that were not identified by Turkers.

The researchers cleaned this data manually, to eliminate false positives. Turkers identified many documents as social media guidelines that were instead documents of several different types:

- Minutes from meetings in which social media was discussed, often meetings of Boards of Governors;
- Pages providing links to all of the social media accounts maintained by the institution;
- Presentations about social media and developing social media policies;
- Course catalogs that list courses that address social media, or the syllabi for such courses; and
- Job postings for staff positions with social media expertise, often in Offices of Communications.

### Stage 3: Content Analysis

The third and final stage of use of Mechanical Turk was to perform content analysis on the social media policy documents, to identify the issues addressed in these documents. This content analysis was conducted between 22 May – 3 July 2013. As discussed above, there has to date been only one content analysis of social media policies [24], though the National Labor Relations Board provides a detailed legal analysis of a set of employers' social media policies [18]. Based on the results of those two analyses, we developed a questionnaire to enable Turkers to conduct content analysis of institutions' social media policies. Prior to launching the content analysis, this questionnaire was piloted twice, with 5 Turkers conducting content analysis on 10 randomly-selected institutions’ social media policies per pilot round. Amazon's Requester Best Practices Guide for Mechanical Turk [33] states that tasks should be kept simple and short, and our pilot bore this advice out: the questionnaire was separated into two parts, to reduce the size of the task. Also on the basis of the pilot, the questions on the questionnaire were simplified, reducing the content analysis to a series of mostly multiple-choice and yes/no questions (e.g., “Which specific websites or services are mentioned by name, if any? (Check all that apply): Facebook, Twitter, LinkedIn, etc.” and “Does the policy discuss copyright? Yes / No”).

Content analysis often involves more than multiple-choice and yes/no questions. But content analysis can also be used to quantitatively describe the content of texts [34] [35], essentially turning content analysis into a categorization task. Categorization tasks are apparently quite common on Mechanical Turk, because Turk provides a “Categorization App”: that is, a template for creating categorization tasks, along with some standardized controls over the Turkers' workflow. We used this template in developing our content analysis questionnaire.

In any content analysis task, it is important to compute a measure of inter-coder reliability. This was especially important in this study, given the large number of Turkers who participated as coders, and the fact that these coders, being anonymous, could not communicate either with the researchers or each other during the task to discuss their disagreements and solidify the operationalization of the issues addressed in policy documents. This operationalization was performed by the researchers, which is why the content analysis questionnaire was piloted twice: to allow ample opportunity for Turkers to find issues with the operationalization, and to provide the researchers more opportunity to simplify the questionnaire. A large number of Turkers participated as coders throughout this task, and each social media policy document was coded by three Turkers. Krippendorff’s alpha [36] was therefore used as the measure of inter-coder reliability, as, unlike many agreement statistics, it may be used to calculate the agreement between more than two coders. Krippendorff’s alpha was calculated for each question on the content analysis questionnaire; due perhaps to the simplicity of the questions, values for alpha were quite high, ranging from 0.96 for the question, “Which specific websites or services are mentioned by name, if any?” to 0.78 for the question “Does the policy discuss how to post about events at the institution?”

## Results

### Types of Institutions with Social Media Policies

After removing false positives, 822 institutions were found to have social media policies (17.7% of institutions in the Carnegie Classifications Data File, or 22.7% of the 3,620 unique institutions). Of these, 660 institutions had policies only for the institution, 91 had policies only for one or more individual departments or campus units, and 71 had policies both for the institution and for one or more departments or units (80.3%, 11.1%, and 8.6% of institutions with social media policies, respectively).

Institutions in each top-level category in the Carnegie Classifications Data File were found to have social media policies at the following rates:

- Doctorate-granting Universities: 50.3%
- Master's Colleges and Universities: 32.1%
- Baccalaureate Colleges: 16.4%
- Associate's Colleges: 8.7%
- Special Focus Institutions: 8.6%

**Fig 1:**
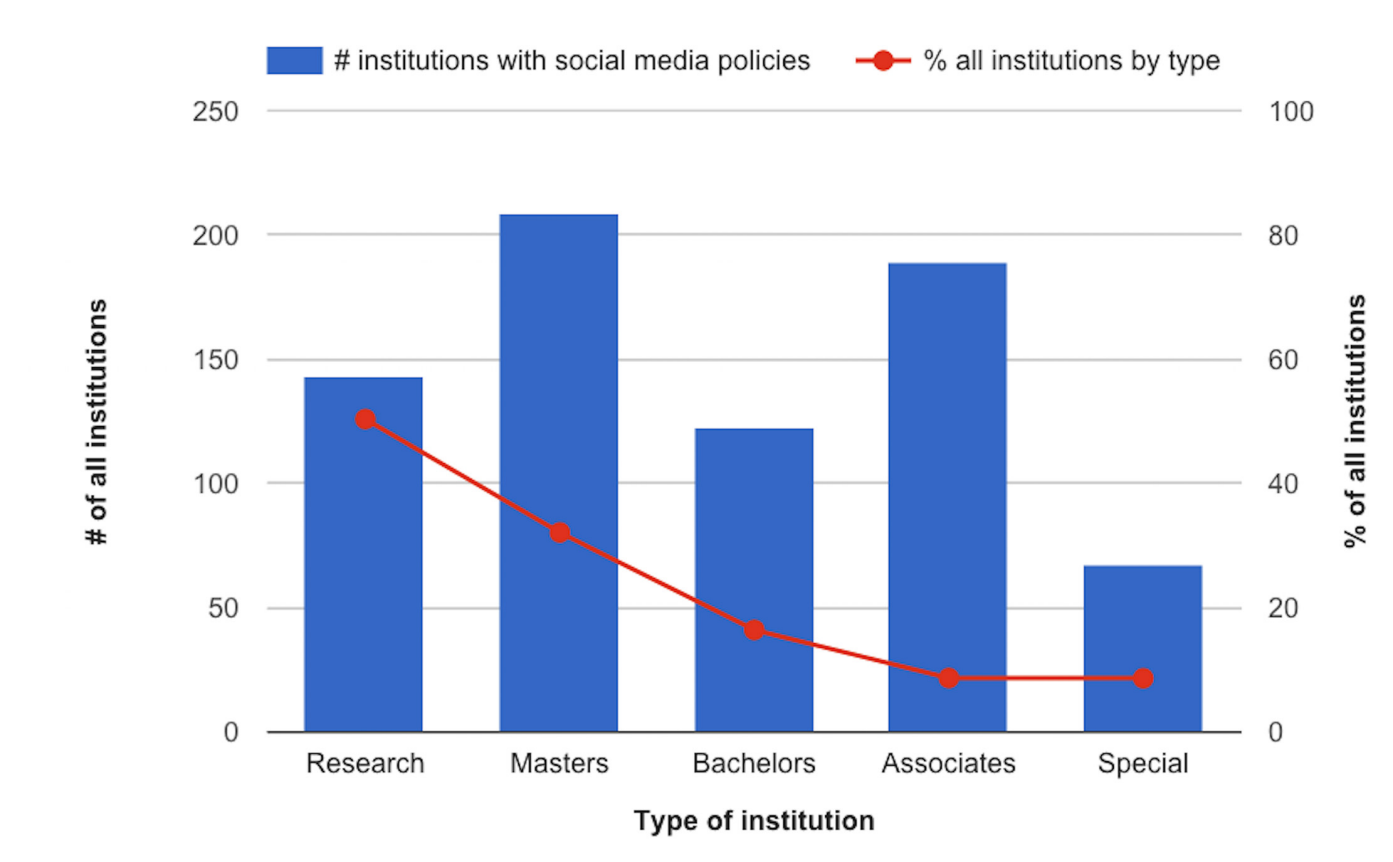
Number and percentage of institutions with social media policies, by Carnegie Basic Classification category.

Fig 1 shows (1) the total number of institutions of each Carnegie Basic Classification category that have social media policies of any kind and, (2) those numbers as percentages of all institutions of those categories according to the Carnegie Classifications Data File. Associates institutions compose far and away the greatest percentage of institutions in the Carnegie Classifications Data File, at nearly 47% of all institutions in the US. However, more Masters' institutions than any other type have social media policies (Masters' institutions comprise only 14% of institutions in the US) and a greater percentage of Research institutions (only 6% of all institutions in the US) have social media policies than any other type.

**Fig 2:**
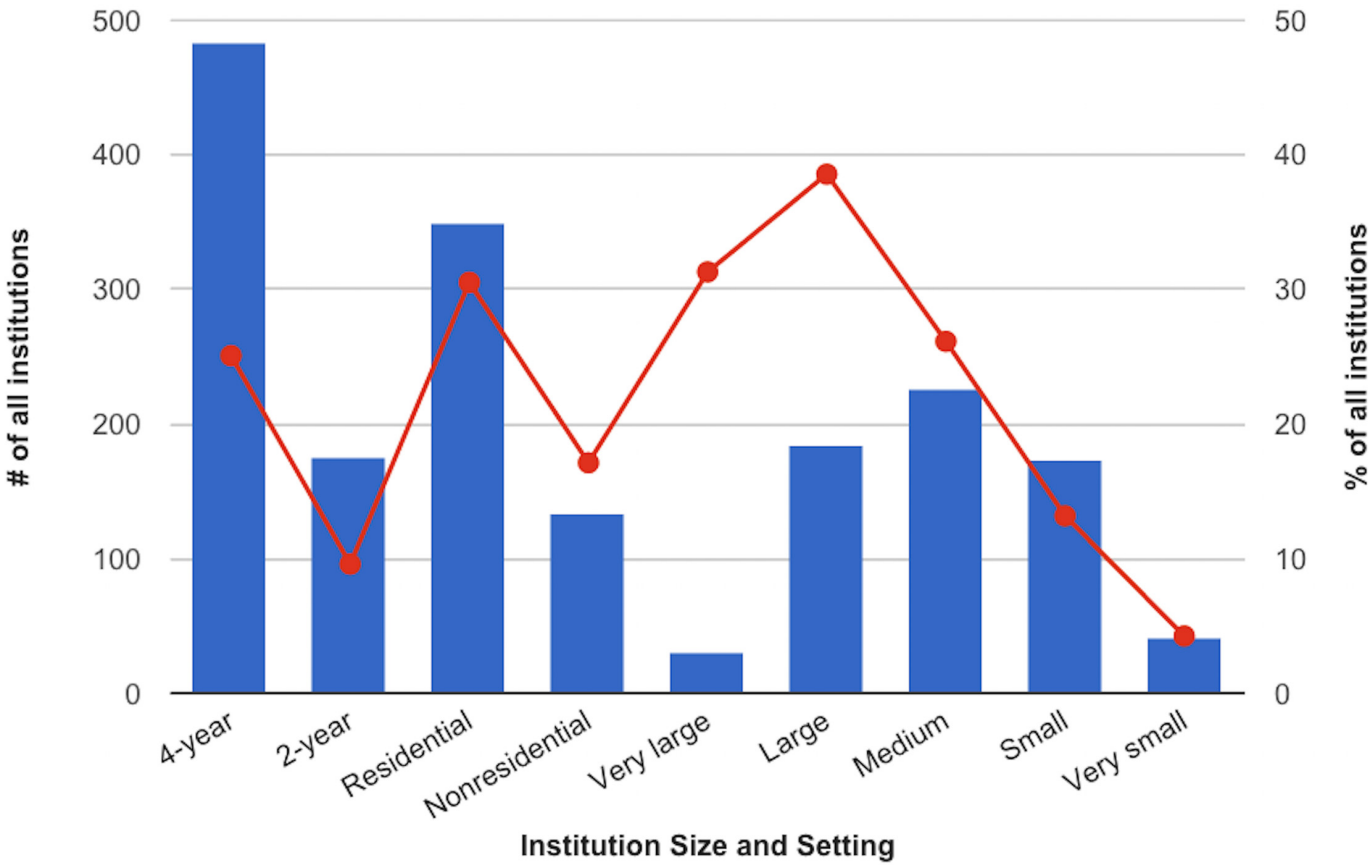
Number and percentage of institutions with social media policies, by Carnegie Size and Setting variable.

The Carnegie Classifications Data File contains a variable, Size and Setting Classification, which combines three factors: whether an institution is 4-year or 2-year, whether an institution is residential or non-residential, and the size of the institution. Institution sizes include Very small (fewer than 500 students for 2-year institutions / fewer than 1,000 students for 4-year institutions), Small (500–1,999 students for 2-year institutions / 1,000–2,999 students for 4-year institutions), Medium (2,000–4,999 / 3,000–9,999), Large (5,000–9,999 / 10,000 or more for 4-year institutions), and Very large (10,000 or more for 2-year institutions). The Size and Setting Classification variable combines these factors for a total of 21 subcategories, but for this analysis, these three factors have been split out. Fig 2 shows the percentage of all institutions of those types that have social media policies.

A far greater percentage of residential institutions have social media policies than non-residential institutions, and a far greater percentage of 4-year institutions have social media policies than 2-year institutions. There is, of course, a strong correlation between the number of years of an institution and its residential status: The Associate degree is a 2-year degree, which is largely (though not exclusively) the degree offered by community colleges, and community colleges are largely (though not exclusively) non-residential. So, while both non-residential 4-year and residential 2-year institutions exist, to a certain extent the findings that 4-year and residential institutions are more likely to have social media policies than 2-year and non-residential, are two faces of the same finding.

No other breakdown of institutions by categories articulated in the Carnegie Classifications Data File showed any notable differences. Several analyses were conducted: public/private control of the institution; enrollment profile (exclusively undergraduate, exclusively graduate, mixed, etc.), geographic region (Southeast, Great Lakes, etc.); and accrediting agency (Southern Association of Colleges and Schools, North Central Association of Colleges and Schools, etc.), which of course also loosely corresponds to geographic region.

### Campus Units with Social Media Policies

Large institutions were more likely than any other size to have individual units with their own social media policies: 15.7% of all unique large institutions, but 5% or less for institutions of all other sizes. This is likely due to the fact that large institutions are more likely than smaller to be divided up into units that operate semi-independently, such as colleges, schools, or medical facilities. The fact that “very large” institutions did not have policies at the same rate is likely an artifact of the Carnegie Classification data file, as very large institutions are exclusively 2-year institutions – and as discussed above, 2-year institutions are less likely to have social media policies.

There was a great deal of consistency in the individual campus units at institutions that have social media policies. Nursing Departments were consistently the most likely to have policies, with 26% of all social media policies at the unit level referring to these departments. Counting all medical- and health-related units (e.g., Pharmacy, Dental Hygiene, Radiology, etc.), this figure is 54%. Athletics and campus Libraries came in a distant second and third, at 10% and 7% respectively.

No other analyses of institutions that have individual units with their own social media policies showed any notable differences.

### Content Analysis of Social Media Policies

As discussed above, content analysis was performed on the social media policy documents, to identify the issues addressed in these documents.

**Fig 3:**
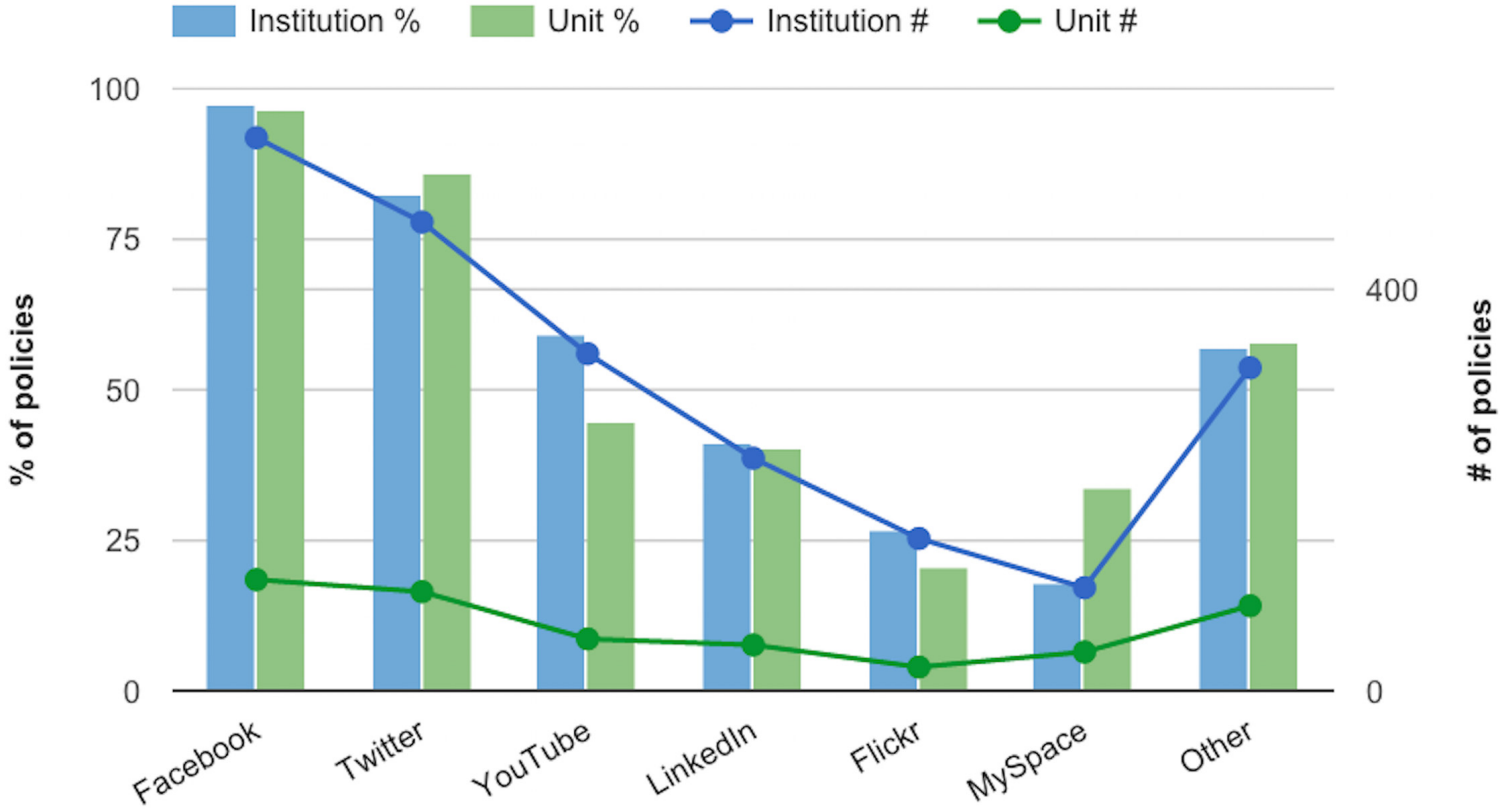
Web services addressed in social media policies.

Fig 3 shows the web services that are addressed by name in social media policies. Facebook and Twitter are the most frequently mentioned: Facebook is nearly ubiquitous, being mentioned in 97.5% of policies for the institution, and 96.5% for the unit. Twitter was a close second at 82.6% and 86.1%, respectively. The Other category includes all services listed in less than 10% of policies. These include: blogs (8.3%); Wikipedia (7.4%); Pinterest (6.6%); FourSquare (6.2%); and Instagram, Tumblr, iTunes and iTunesU, and Vimeo, all at under 5% each. The percentages in Fig 3 do not sum to 100%, because a single policy document may mention several social networking services by name.

**Fig 4:**
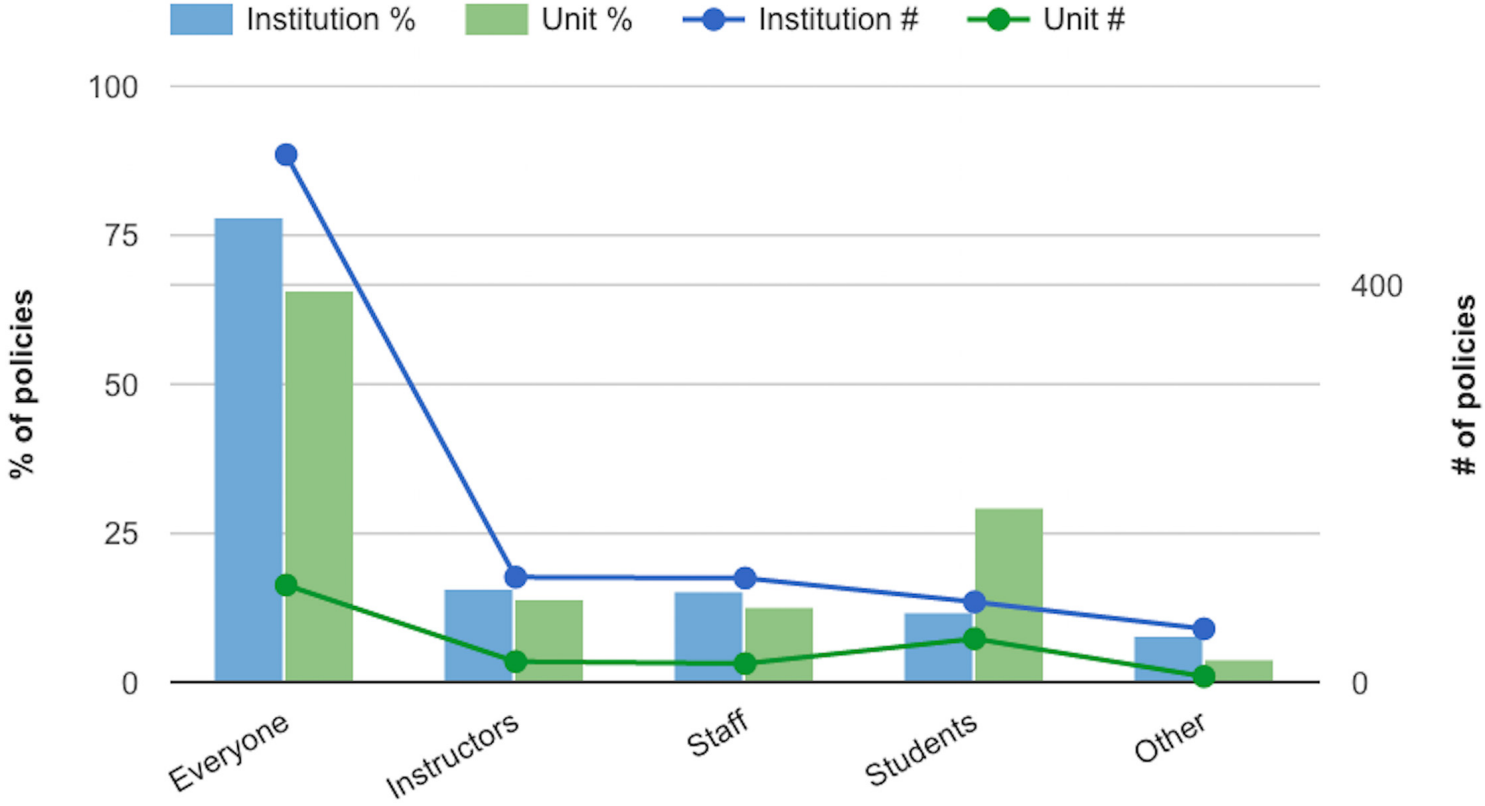
Communities for whom social media policies are written.

All policies are written to apply to entire communities. However, many identifiably distinct communities exist at institutions of higher education. Fig 4 shows the communities that are addressed by name in social media policies. Most social media policies, both for institutions and for individual units, are written to apply to all members of the relevant community. However, more social media policies for individual units than for institutions, are written to apply to students. As mentioned above, the majority of campus units with their own social media policies are medical- and health-related; these policies therefore address the behavior of medical practitioners-in-training. Given the protections for patient information in the Health Insurance Portability and Accountability Act of 1996 (HIPAA), it makes a great deal of sense that health-related units would develop policies to ensure that their students are aware that these protections apply to social media.

The Other category in Fig 4 includes all other communities on campus for whom a social media policy may be relevant: alumni, athletics departments, and social media professionals.

Social media policies, like many policies, often refer to other related policies. The social media policies for institutions referred to other policies that you might expect: codes of conduct (23%), copyright and intellectual property policies (23%), policies regarding acceptable use of technology (21%), and privacy policies (14%). Almost no social media policies for campus units referred to other policies.

As discussed above, the content analysis performed on social media policies was based on the results of analyses by Boudreaux [24] and the National Labor Relations Board [18]. Based on the results of those two analyses, we developed a questionnaire to enable Turkers to identify topics addressed in social media policies.

**Fig 5:**
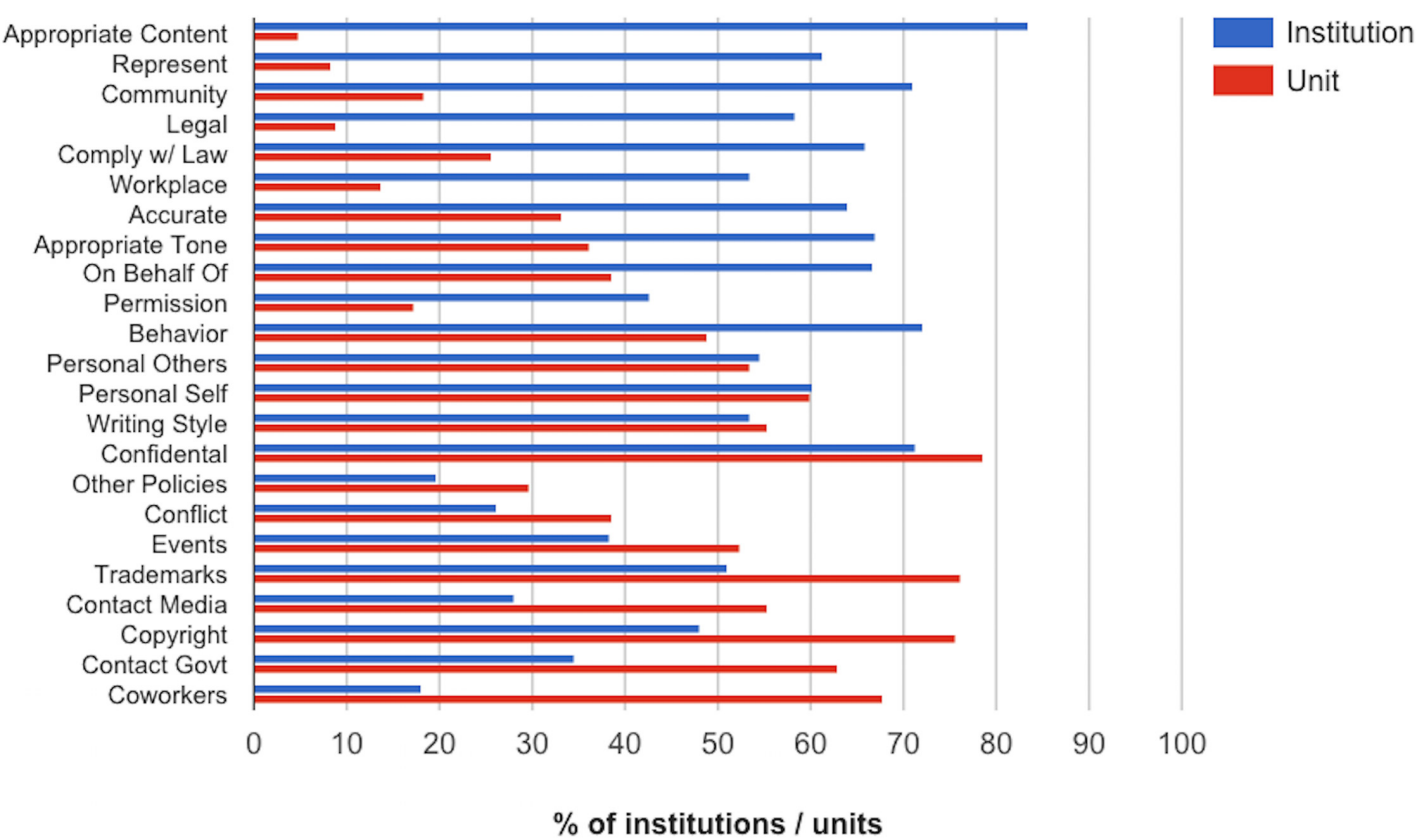
Topics addressed in social media policies.

To assist in reading Fig 5, please note that it is arranged as follows: topics addressed in more policies for institutions than units are on the top, topics addressed in more policies for units than institutions are on the bottom, and the topics are arranged along the vertical axis in order of difference between the institution and the unit.

A large number of topics were addressed in social media policies, but these can be grouped together into several categories. Social media policies for institutions addressed 3 categories of topics more often than policies for units: the appropriateness of posts (e.g., appropriate content, appropriate tone), representing the institution (e.g., branding, public image, posting in the institution’s name), and ensuring that posts comply with the law. Social media policies for units addressed 2 categories of topics more often than policies for institutions: communication between coworkers, and contact with external agencies (e.g., the media, state and local government). Table 1 shows some examples of policies in these categories.

**Table 1:** Example social media policies in various categories.

Appropriateness of posts	Representing the institution
Appropriate content:	Posting in the institution’s name:
“Never give banking information out over social networking and inform students of this policy as well. If it is required specifically for a project this should be a guided process.”	“Never represent yourself or [the institution] in a false or misleading way.”
“Do not reveal the personal health information of individuals that you access in your professional role. This is considered a HIPAA violation.”	“Any messages that might act as the ‘voice’ or position of [the institution] or a college unit must be approved by [the institution] or the director of the college unit or their delegate.”
Appropriate tone:	Public image:
“Be respectful. You are more likely to achieve your goals or provoke thoughtful discussion if you are constructive and respectful while discussing a bad experience or disagreeing with a concept or person.”	“[The institution]’s name shall not be used to promote a product, cause, political party or candidate.”
Communication between coworkers:	Branding:
“Be respectful to [the institution], other employees, students, and other related institutions.”	“Logos, trademarks or any other images from [the institution] may not be used without prior approval.”
“Make social media a part of your workflow. Tell your supervisor, co-workers and counterparts that you’re incorporating social media into your work.”	Ensuring that posts comply with the law:
	“Laws, ethics, and behavior expectations that govern professional life apply equally when posting content on behalf of any of [the institution]’s functional units.”
	“Remember that you are legally responsible for anything you post online. Ensure you abide by copyright and fair use laws. Always cite sources and references and, whenever possible, link back to them.”
	Contact with external agencies:
	“Media contacts about [the institution] should be referred for coordination and guidance to the President’s Office.”

The content analysis investigated whether any social media policy documents addressed the National Labor Relations Board report concerning social media. Despite the fact that memorandum OM 12–59 [18] was written to address “employers’ policies… unlawful under the National Labor Relations Act,” only one institution’s social media policy even mentions the NLRA; that one institution was Harvard University.

## Discussion

Our analysis revealed that less than one-quarter of institutions had an accessible social media policy. Our analysis revealed that less than one-quarter of institutions had an accessible social media policy. It is possible that this is an underestimate, and that other — perhaps many other — institutions had social media policies that were inaccessible due to being password-protected or otherwise inaccessible via the open web. The authors consider this unlikely, however, as institutions of higher education generally have their policy and handbook documents accessible via the open web.

Doctorate-granting universities were proportionally more likely to have social media policies than any other institution type. Policies at the unit level were most likely to appear in health-related discipline, in athletics departments, and in libraries. Policies frequently mentioned particular services, with Facebook and Twitter among the most frequently mentioned. Policies typically applied to all those affiliated with the institution and instructed members of the community to post appropriate content, to represent the institution appropriately and to moderate conversations with coworkers and external agencies. This state-of-the-art study provided a depiction of the social media landscape for higher education and insights into the development of this area of research and practice.

### Rapidly Changing Landscape

Institutions of higher education like their policies. Which is why it was something of a surprise that neither New York University nor the University of New Mexico had policies addressing appropriate conduct on social media to employ when considering the case of Geoffrey Miller. When data was collected for this study, no social media policy existed on New York University’s website, and the University of New Mexico had only a draft policy. By the time this paper was completed, however, both universities had finalized policies in place.

Institutions of higher education are not known for the rapidity of their policy development, but speed is a necessity in the world of social media. In order to keep pace with this development, it behooves institutions of higher education to not only develop social media policies (or to integrate social media into other policies, such as honor codes and codes of conduct), but to revisit them frequently, as the applications and uses of social media evolve. This “policy gap” is especially evident in the finding that many social media policies mention MySpace by name, despite its decreasing popularity [37], while few mention Instagram or Tumblr, despite their growing popularity.

It could be argued that it is a more sophisticated approach to social media for an institution to not have a social media policy [38] – for an institution to instead have policy documents that address social media in the context of broader policies addressing conduct and behavior. The existence of a separate social media policy may be an indication that an institution is still coming to grips with the functionality and affordances of this technology, and is in the process of figuring out how to integrate social media into its culture and practices. The existence of policies that address social media as part of campus culture may be an indication that social media is better integrated into institutional culture; such policies may be “sufficiently flexible to withstand future developments in technology and the endless creativity of its misusers” (p.5). However, whether an institution has a standalone or integrated policy, the openness and transparency of the communication space, and the changes wrought by this transformation, must be taken into account [39].

### Enforceability and Interoperability

As can be seen in the examples in Table 1, some social media policies referred to individuals or campus units (e.g., “the director of the college unit,” “the President’s Office”) to which questions or other issues should be referred. But none referred to any individuals or campus units that had authority to enforce these social media policies.

As mentioned above, a far greater percentage of residential institutions have social media policies than non-residential institutions, and a far greater percentage of 4-year institutions have social media policies than 2-year institutions. It may be simply that in an intentional community such as a residential campus setting, there is more of a need for policies regulating behavior to be explicit, while at non-residential institutions students’ behavior is more strongly influenced by factors off-campus, such as work and family. Similarly, very large, large, and medium institutions are more likely to have social media policies than small and very small institutions. This may reflect the social dynamic whereby interpersonal social norms are often a stronger force in smaller communities, while explicit policies often must be used to combat the anonymity of larger communities, and enforce those same social norms.

Large institutions commonly have individual units with their own social media policies. This makes sense, as large institutions are often divided up into units that operate semi-independently, such as colleges, schools, or medical facilities. These units have communities that are of course smaller than the campus community as a whole, but may still be quite large. We did not find any policies from campus units that were obviously in conflict with policies for the institution as a whole (at those institutions that had both), but this is clearly a risk that should be considered in the development of macro- and micro-level policies.

As discussed above, Boudreaux [24] identified three distinct stages in the evolution of social media policies: Mitigation, Information, and Differentiation. Many social media policies in institutions of higher education are, at present, in the Mitigation phase of evolution, with some beginning to enter the Information phase. Many policies are remarkably similar, containing advice on the proper “voice" to use on social media, respect for others, representing the institution, copyright, and other topics that apply equally to any institution of higher education. Some institutions have started to move beyond this simple advice, to use the social media policy as a vehicle for disseminating information, such as links to other relevant policy documents. By and large, however, there are few notable differences between policies at different institutions, and even few differences between policies for different campus units at different institutions. The most significant difference is that social media policies for medical- and health-related units on campus address the Health Insurance Portability and Accountability Act of 1996 (HIPAA), where policies for non-health-related units do not. To be fair, however, there is little differentiation across institutions of higher education in other types of policies, such as student honor codes [40].

Some institutions integrate social media policies into other policy documents, such as student honor codes, and faculty and student codes of conduct. Some of these policy documents explicitly address social media: this following policy, for example, was taken from a section on social media in a document concerning all institutional policies:

“Social media usage at [institution] is governed by the same policies that govern all other electronic communications.”

Similarly, this policy was taken from a student handbook:

“for students that access these sites on personal computers or phones, [institution] has the expectation that students will uphold the ethical standards of their prospective professions and the [institution] Student Code of Conduct.”

On the other hand, some policy documents do not mention social media at all, implicitly treating it as just another forum for interpersonal interaction, as such falling under the jurisdiction of existing policies. For example, the following policy was taken from a student honor code document:

“The activities of students, as well as other members of the [institution] community outside the classroom, influence the educational process and learning environment, just as the intellectual atmosphere of the campus contributes to students’ growth and development. Many forms of nonacademic conduct, as well as all facets of the academic process, are therefore areas of proper concern and regulation by the [institution] community.”

It was somewhat surprising that accrediting agency did not correlate with the existence or nonexistence of social media policies, since accrediting agencies have input into so many aspects of the operation of institutions of higher education, including policies. However, this finding (or lack thereof) indicates that the development of social media policies is, at present, idiosyncratic and institution-specific, and has not yet been integrated into the culture of higher education broadly.

### Academic Freedom

The issue with perhaps the greatest degree of complexity in drafting social media policies is finding language that simultaneously protects the reputation of the institution while respecting academic freedom and First Amendment rights. The social media policy constructed by the Kansas Board of Regents [41] in response to the heavily discussed tweet of a University of Kansas professor includes the following quote from the 1940 Statement of Principles on Academic Freedom and Tenure of the American Association of University Professors [42]:

“College and university teachers are citizens, members of a learned profession, and officers of an educational institution. When they speak or write as citizens, they should be free from institutional censorship or discipline, but their special position in the community imposes special obligations. As scholars and educational officers, they should remember that the public may judge their profession and their institution by their utterances. Hence they should at all times be accurate, should exercise appropriate restraint, should show respect for the opinions of others, and should make every effort to indicate that they are not speaking for the institution.”

The Kansas Board of Regents used this as justification for censuring faculty members on social media platforms, particularly when these faculty members self-identified as a member of the institution or otherwise evoked the name of the institution on the social media platform. In response, the AAUP condemned the Kansas social media policy “as a gross violation of the fundamental principles of academic freedom” [43] (para. 1). Future policies will need to navigate the balance between academic freedom and institutional branding with care.

## Conclusion

Future research – both in the academy and outside of it – is needed to investigate characteristics of organizations with social media policies in Boudreaux’s [24] three stages, and to investigate how an organization might progress more rapidly through those stages. Guidelines are also necessary for institutions struggling with the construction of social media policies. However, academic institutions are not alone in this endeavor. Administrators and policy makers may want to familiarize themselves with the National Labor Relations Board’s [18] reports on social media policies. These reports were borne out of the NLRB’s finding that some social media policies from the business sector contained provisions that were not legal. These reports articulate examples of illegal provisions, but more importantly, examples of what the NLRB suggests as best practices. Institutions of higher education would be wise to take note.

This study is descriptive of the “state of the art” in the development of social media policies in higher education, in 2013. Like all reports on states of the art, this one is likely to become rapidly dated. In the time it has taken the authors to write this paper, several stories about issues surrounding social media in institutions of higher education have been in the news, including those about East Stoudsburg University and the University of Kansas, mentioned above. In institutions of higher education, this sort of policy development often takes place in committees, and the second stage of data collection in this study found several committee meeting minutes in which the development of social media policies was discussed. There is, however, no way to know how many other institutions may also be in the process of developing social media policies. Given the potential for liability, and the National Labor Relations Board’s attention to social media policies, it behooves institutions of higher education to develop policies quickly that take into account the rights of students and faculty, while mitigating risk to the institution.
